# The Code of Dental Ethics

**Published:** 1867-10

**Authors:** 


### EDITORIAL DEPARTMENT.

*The Code of Dental Ethics.*—We find in the September No. of the *Dental Register* the following notice:

"During the Exhibition of Instruments and Appliances at the meeting of the American Dental Association, held in Cincinnati a few days ago an Automatic Plugging Instrument passed in an illegitimate way from us to the possession of some other person. That person is known, and if he wishes to avoid any further publicity of the matter, he will return the instrument at once, and nothing more will be said about it. If it does not come home we will take measures to bring it home. We don't like that style of borrowing."

Several other articles on exhibition, besides the one referred to in the notice, we also learn, "passed in an illegitimate way" from their owners to some other persons; and yet this Association at its late meeting refused to adopt the *Code of Dental Ethics*, prepared by a committee appointed for the purpose, at the Boston meeting in 1866. Earnest efforts were in vain made by several influential members at the late meeting, to have this or some other code adopted, which would guard against the admission of unworthy members.

Such acts as we refer to will, we think, convince the most skeptical of the necessity existing for the adoption of stringent rules governing the admission of members and delegates. Article II. of the rejected Code refers to the maintaining of professional character, and reads as follows:

Sec. 1. A member of the dental profession is bound to maintain its honor, and to labor earnestly to extend its sphere of usefulness. He should avoid everything in language and conduct calculated to dishonor his profession, and should ever manifest a due respect for his brethren. The young should show special respect to their seniors; the aged special encouragement to their juniors.

Sec. 2. The person and office arrangements of the dentist should indicate that he is a gentleman; and he should sustain a high-toned moral character.

Sec. 3. It is unprofessional to resort to public advertisements, cards, handbills, posters, or signs calling attention to peculiar styles of work, lowness of prices, special modes of operating; or to claim superiority over neighboring practitioners; to publish reports of cases or certificates in the public prints; to go from house to house to solicit or perform operations; to circulate or recommend nostrums; or to perform any other similar acts.

Sec. 4. When consulted by the patient of another practitioner, the dentist should guard against inquiries or hints disparaging to the family dentist, or calculated to weaken the patient's confidenee in him; and if the interests of the patient will not be endangered thereby, the case should be temporarily treated, and referred back to the family dentist.

Sec. 5. When general rules shall have been adopted by members of the profession practicing in the same localities in relation to fees, it is unprofessional and dishonorable to depart from those rules, except when variation of circumstances requires it. And it is ever to be regarded as unprofessional to warrant operations or work, as an inducement to patronage.

